# Does affective touch buffer emotional distress? Insights from subjective and physiological indices

**DOI:** 10.1093/scan/nsaf090

**Published:** 2025-09-05

**Authors:** Letizia Della Longa, Michela Sarlo, Teresa Farroni

**Affiliations:** Dipartimento di Psicologia dello Sviluppo e della Socializzazione (DPSS), University of Padova, Padova, 35131, Italy; Dipartimento di Scienze della Comunicazione, Studi Umanistici e Internazionali (DISCUI), University of Urbino Carlo Bo, Urbino, 61029, Italy; Dipartimento di Psicologia dello Sviluppo e della Socializzazione (DPSS), University of Padova, Padova, 35131, Italy

**Keywords:** affective touch, self-regulation, infant crying, heart rate variability, stress index

## Abstract

Affective touch, mediated by the activation of C-tactile afferents, has the potential to modulate affective states and physiological responses in situations of emotional distress across the lifespan. The present study aims to disentangle psychophysiological mechanisms supporting autonomic and emotional self-regulation, focusing on the possible buffering role of affective touch. Childless adult participants (*N* = 92) were presented with videos of an infant babbling (positive scene) and an infant crying (emotionally negative scene), followed by a tactile stimulation that was either affective (brushing) or non-affective (tapping). We collected subjective measures of affective state (valence and arousal) and physiological measures: heart rate (HR), HR variability (root mean square of successive differences [RMSSD] index), and stress index (SI). Participants reported a decrease in pleasantness and an increase in arousal during the crying video. Moreover, participants receiving affective touch showed an overall greater increase in pleasantness. At the physiological level, tactile stimulations elicited HR deceleration, reduction of SI, and return to baseline levels of RMSSD after emotional activation. These responses were more prominent in the affective touch group, suggesting that affective touch is effective in decreasing sympathetic activity and increasing vagal dominance. Our results indicate that affective touch may play a central role in autonomic and affective regulation, possibly buffering emotional distress.

## Introduction

Interpersonal affective touch acts as an external regulator of emotional arousal and physiological regulation during situations of distress across the lifespan (Cascio et al. 2019, Carozza and Leong 2021, Farroni et al. 2022, Kidd et al. 2023). Previous evidence elucidated the efficacy of touch intervention on physical pain and stress in both developmental and adult population, suggesting a significant role of touch in buffering neural, physiological, and psychological effects of stress (Coan et al. 2006, Holt-Lunstad et al. 2008, Packheiser et al. 2024). Such effects seem to be specifically mediated by the social-affective dimension of touch, known as affective touch, which is characterized by positive affective value, lower arousal, and modulation of neural responses (Walker et al. 2022a, 2022b). The encoding of affective touch begins in the skin, where a class of low-threshold mechanoreceptors, the C-tactile (CT) afferents, select and encode tactile information that is most likely to carry socio-emotional information (Morrison et al. 2010). The firing rate of CT afferents increases in response to dynamic tactile stimulation delivered at low force (range 0.3–2.5 mN; Vallbo et al. 1999), slow velocity (1–10 cm/s; Löken et al. 2009, McGlone et al. 2014), and skin temperature (around 32°C; Ackerley et al. 2014), and correlates with subjective feelings of pleasantness (Löken et al. 2009). The information from CT afferents is conveyed in a specific somatosensory pathway that constitutes an interoceptive submodality signalling the emotional and social relevance of interpersonal tactile interactions (Olausson et al. 2010). At the neural level, CT-optimal affective touch activates a network of brain areas related to socio-emotional processing, including the anterior cingulate cortex, superior temporal sulcus, orbitofrontal cortex, medial prefrontal cortex, amygdala, hippocampus, and hypothalamus (Gordon et al. 2013, Sailer et al. 2016, Morrison 2016a, Kirsch et al. 2020, Meijer et al. 2022). At the physiological level, several studies documented positive biochemical and autonomic effects of touch, including decreased cortisol levels—a hormone related to stress reactivity—and increased oxytocin levels—related to social bonding and affiliative behaviours (Schneider et al. 2023) as well as decreased blood pressure and modulation of cardiac activity (Field 2010, Kidd et al. 2023). Specifically, stimulation of CT afferents within an optimal stroking velocity range has been shown to elicit a decrease in heart rate (HR; Fairhurst et al. 2014, Pawling et al. 2017b) and an increase in heart rate variability (HRV; Triscoli et al. 2017, Van Puyvelde et al. 2019). These changes in cardiac dynamics and HRV, modulated by input from CT‑afferents and their integration within spinal and central circuits, highlight a ‘skin-heart’ interplay, suggesting that affective touch may have a significant impact on autonomic regulation.

Within the framework of motivated emotion theory (Lang and Bradley 2010), affective touch targeting CT afferents is associated with positive affective states low in arousal, signalling reassurance, comfort, and safety and acting as an appetitive motivational stimulus (Walker et al. 2022a). It has been hypothesized that a brief affective touch activates an appetitive motivational reflex, characterized by an initial vagal-mediated HR deceleration followed by a brief acceleration that functions to increase attention and facilitate perceptual processing (i.e. an orienting response); while a sustained CT-targeted affective touch results in decreased sympathetic activity and increased parasympathetic dominance (Morrison 2016b), buffering against the behavioural and physiological consequences of stress, as suggested by animal models (Walker et al. 2022b).

Despite numerous studies reporting consistent modulations of cardiac activity during tactile stimulation, the complex interplay between sympathetic and parasympathetic contributions to the modulation of cardiac dynamics has been rarely explored in the context of tactile stimulation (Candia-Rivera et al. 2024). Initial evidence suggests that inhibition of sympathetic activity may enhance the sensitivity of tactile receptors through ascending signalling from the skin to the brain via spinal cord pathways (Kissin et al. 1987). Recent work has highlighted that affective touch engages distributed brain–body networks, including brain–spine and brain–heart circuits that integrate peripheral sensory input with interoceptive signals and top-down modulation. For example, somatosensory-evoked potentials have been shown to differentiate the bodily self from others, with reduced amplitudes at the cortical level during self-touch and faster latencies, both at the cervical spinal and cortical levels, during other-touch, indicating that top-down signalling can be integrated with and modulate early sensory processing within brain-spine networks (Boehme et al. 2019). Moreover, social touch, compared with self-touch, was found to enhance coupling between fronto-parietal networks and vagal activity (as indexed by HRV), while reducing coupling with sympathetic dynamics (Candia-Rivera et al. 2025). This suggests that cardiac signals are integrated within brain–heart networks, where top-down modulation shapes the cortical processing of affective touch.

According to the neurovisceral integration model (Thayer and Lane 2000, 2009, Thayer and Siegle 2002, Thayer and Brosschot 2005), the autonomic nervous system (ANS) modulates and integrates both attentional and affective components, providing a neurophysiological basis for psychological health, which can support our understanding of emotion regulation and dysregulation. The balance between parasympathetic (vagal) and sympathetic branches of the ANS functions in the service of self-regulation and adaptability of the organism to the dynamically changing environment (Chu et al. 2024). This model suggests a bidirectional feedback loop between the central nervous system (CNS) and the cardiovascular system mediated by the vagus nerve, which detects increases in physiological arousal and inhibits this activity by slowing the HR and increasing the vagal tone to restore energy resources (Heiss et al. 2021). Beyond homeostatic regulation, bidirectional communication and influence between the CNS and ANS mediated by interoceptive neural circuits play a crucial role in shaping emotional experiences (Cai et al. 2024). Subjective feelings may emerge from ascending interoceptive signals to the brain as a consequence of functional brain–heart interplay (Candia-Rivera et al. 2022). Additionally, spinal circuits should be considered as potential pathways involved in the sympathetic deactivation observed during affective touch. CT primary afferents enter the dorsal horn of the spinal cord, where interneuronal circuits integrate tactile inputs with descending inputs from supraspinal centres before transmitting them to ascending projection neurones. These integrative circuits can influence the excitability of sympathetic preganglionic neurones in the intermediolateral cell column, thereby modulating sympathetic outflow (e.g. Deuchars and Lall 2015). Synergistic brain–heart interactions, involving both sympathetic and parasympathetic modulations, are essential for adaptive emotional responses, flexibility in the face of environmental changes, and effective coping with stress throughout development (Norman et al. 2014, Faig et al. 2023). Employing multiple cardiac indices may offer deeper insights into the complex regulation of cardiac dynamics in relation to sensory and emotional processes.

In the present study, three indices of cardiac activity have been considered: HR, the squared root of the mean squared differences between successive heart periods (root mean square of successive differences [RMSSD]), and the stress index (SI). HR can be easily computed from the inter-beat interval (IBI) series, expressed in beats per minute (bpm), and is the result of the interaction between parasympathetic (vagal) and sympathetic activation (Draghici and Taylor 2016). HR changes may be related to motor activity and bodily movement (cardiac acceleration to fulfil metabolic needs), activation of the sympathetic system in response to stressors or emotional stimuli (cardiac acceleration as an arousal-related response), or cognitive and attentional processes (cardiac deceleration to facilitate sensory information processing; Stern et al. 2000). Among HRV indices, the RMSSD is particularly relevant, as it captures short-term variability in interbeat intervals and is considered a reliable marker of parasympathetic cardiac activity (McCraty and Shaffer 2015, Draghici and Taylor 2016, Shaffer and Ginsberg 2017). Beyond these extensively used indices, the HRV-derived SI, computed by the Kubios software as the square root of Baevsky’s SI, reflects sympathetic activation during mental, emotional, or physical stress (Baevsky and Chernikova 2017, Sahoo et al. 2019). Baevsky’s SI reflects cardiac rhythm stabilization in response to mental or physical stress, with a decrease in the range of IBI durations and an increase in the number of IBIs of similar duration, suggesting stronger sympathetic activation and reduced vagal modulation. While not regarded as a standard HRV parameter, this measure has been increasingly used as an index of stress-related autonomic regulation in both healthy and clinical populations (Ognev et al. 2019, Brisinda et al. 2024, Huizinga et al. 2025). The use of multiple indices is crucial for assessing balance in autonomic regulation, considering activity in both the sympathetic and parasympathetic branches of the ANS, to better understand the psychophysiological responses of activation and relaxation.

In the present study, we employed subjective measures of affective state and multiple indices of cardiac activity during the presentation of emotionally salient videos, followed by tactile stimulation, with the aim of disentangling psychophysiological mechanisms supporting autonomic and emotional self-regulation, possibly modulated by affective touch. The present investigation offers several strengths and novel contributions, including highlighting both the importance and the challenges of integrating different physiological and subjective measures in a multisensory emotional context; examining the effects of tactile stimuli delivered after emotional activation rather than in isolation; and employing a rigorous and replicable experimental design, making it suitable for other developmental populations.

For these purposes, we presented young childfree adults with two videos depicting an infant babbling (positive affective valence) and an infant crying (negative affective valence). Infant crying represents an ecologically valid and salient affective signal that can elicit strong emotional reactions in other babies (Dondi et al. 1999) as well as in the general adult population, not limited to parents (Cohen-Bendahan et al. 2014, Hechler et al. 2015, Bartlett and McMahon 2016). After the video presentation, participants were presented with tactile stimulation, either affective touch (CT-optimal stroking, called the affective touch group) or non-affective touch (CT-suboptimal tapping, called the non-affective touch group). We expect that the different emotional content of the videos will elicit a modulation of participants’ affective state at both the subjective level, as modulation of valence and arousal, and at the physiological level, as modulation of cardiac activity. More specifically, we anticipate that the emotionally salient scene (infant crying) will elicit more negative valence and increased arousal in respect to the positive scene (infant babbling). We also hypothesize that such pattern of subjective responses will be accompanied by an increase in sympathetic activity, as signed by increases in HR and SI, and a decrease in vagal tone, as indexed by HR variability (RMSSD index; Wu et al. 2019). Critically, we expect that affective touch would be more effective than non-affective touch in restoring the subjective and physiological affective states to baseline levels after the video exposure, as reflected by an interaction effect with the group factor (affective vs non-affective group; Morrison 2016b, Walker et al. 2022a, 2022b).

## Materials and methods

### Participants

Ninety-eight young childfree adults took part in the experiment. Data from five participants were discarded from analysis due to invalid physiological data (e.g. ectopic heartbeat, artefacts, etc.). We excluded from analysis one additional participant due to a baseline RMSSD of 117 ms, which is more than 3 SD over the mean of the sample (mean = 38.84 ms, SD = 17.85). Please note that normative baseline RMSSD values for healthy adults are 42 ms (SD = 15; Nunan et al. 2010). The final sample included 92 participants (80 females and 12 males) between 18 and 28 years (mean age 21.78 years, SD = 2.58). After being informed about the study aims, procedure, and methods, all participants signed the consent form. The study was performed in accordance with the ethical standards of the Declaration of Helsinki. The Ethical Committee of Psychological Research (University of Padova) approved the study protocol (code 4970).

### Stimuli and procedure

**Figure 1.**
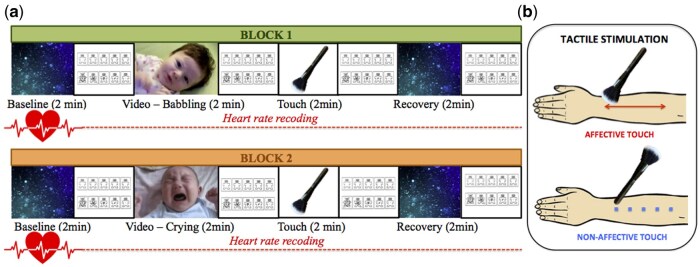
Experimental paradigm displaying the four experimental Phases (Baseline, Video, Touch and Recovery). Panel A: The first phase of each block consisted in presentation of a neutral and silent video (moving babbles) and served as a measure of individual baseline cardiac activity. In the second phase a video of a baby was presented: baby smiling and making some babbling sounds (in block 1), baby crying (in block 2). The third phase consisted in a pause from the video display (black screen) and a tactile stimulation. Finally, the last phase consisted in a recovery period during which the same video used as baseline was presented. Panel B: The affective touch stimulation consisted in a gentle stroking with brush bristles at velocity of approximately 3 cm/s; while the non-affective touch consisted in a gentle tapping with the brush handle at rate of approximately one tap per second.

At the beginning of the experimental session, the experimenter gently positioned three electrodes on the participant’s chest to measure the electrocardiogram (ECG), ensuring participant comfort and compliance. The study consisted of the presentation of two experimental blocks composed of four phases of 2 min each. This interval length has been selected for obtaining accurate measures of HR variability (RMSSD), as indicated by previous studies (Nussinovitch et al. 2011, Munoz et al. 2015, Melo et al. 2018, Pham et al. 2021). We manipulated the emotional valence by comparing a video of a baby babbling and a video of a baby crying, which represents a salient stimulus that has been shown to elicit strong emotional responses not only in parents but also in childfree adults (Hechler et al. 2015). We decided to use videos of infants for two main reasons: first, we were interested in stressing the socio-affective salience of the video, building on the fact that infant emotional expression elicited greater attention compared to adult faces (Thompson-Booth et al. 2014, Gemignani et al. 2024). Second, we aimed to create a research paradigm that could be potentially extensible to other developmental ages. The videos used in this study were adapted from (Ruffman et al. 2019) and were subjectively evaluated by both adults and toddlers (Ruffman et al. 2019). The videos were followed by tactile stimulations applied in a proximal-to-distal direction on the participant’s dorsal forearm (9-cm-long skin area) with a cosmetic brush (5-cm width; see Fig. 1) by a trained female experimenter who sat on the side of the participant to have easy access to the exposed forearm. The tactile stimulation was manipulated as a between-subjects variable. Participants were randomly assigned to two different groups: one group received affective touch (*N* = 43), and the other group received non-affective touch (*N* = 49). The affective touch stimulation consisted of a gentle stroking using brush bristles at low force and a velocity of ∼3 cm/s, while the non-affective touch consisted of gentle tapping with the brush handle at the rate of approximately one tap per second. The two types of touch were selected to match the amount of sensory input (i.e. duration, force, contact area, and stimulation rate), while differing in the affective value conveyed by both the softness (soft brush bristles vs wooden brush handle) and the spatio-temporal dynamics of touch (stroking vs tapping). After each phase of the experiment, participants were asked to report their affective state on the 1–9 point scales of valence (unpleasantness/pleasantness) and arousal (calm/activation) of the Self-Assessment Manikin (SAM; Bradley and Lang 1994). The order of the experimental blocks was fixed by design to prevent possible long-lasting effects of the crying scene. Thus, all participants were first presented with the video of babbling and then with the emotionally salient video of crying (Fig. 1).

### Electrophysiological data recording and processing

The ECG was recorded by means of three Ag/AgCl electrodes placed on the participants’ chests according to Einthoven’s Triangle (Lead II configuration) (Anbalagan et al. 2023). Specifically, we used a multimodality physiological monitoring device that encodes biological signals in real-time (ProComp Infiniti; Thought Technology, Montreal, Canada), which is a computerized recording system approved by the US Food and Drug Administration (FDA). The ECG signal was recorded continuously via a 12-bit analogue-to-digital converter with a sampling rate of 256 Hz and band-pass filtered (0.3–150 Hz). ECG data were imported into the Kubios HRV scientific 4.1.0 software (Oy, Kuopio, Finland), where IBI series were automatically detected using a built-in QRS detection algorithm based on the Pan–Tompkins method (Pan and Tompkins 1985). The IBI series were preprocessed for artefact removal using automatic procedures followed by interactive visual inspection.

### Statistical analyses

All statistical analyses were performed using R, a software environment for statistical computing and graphics (R Core Team 2023). In order to assess whether the experimental manipulations were effective in modulating participants’ subjective affective state, we first analysed the subjective measures of valence and arousal, considering the differential score in respect to baseline levels. Then, we investigated participants’ modulation of cardiac activity by considering the indices of HR, RMSSD, and SI across different phases of the experiment and during affective vs non-affective tactile stimulation. We controlled for potential confounding differences in individual physiological activity by running preliminary analysis on baseline levels of all cardiac indices of interest (HR, RMSSD, SI; Table S1 and Fig. S1, see online supplementary material for a colour version of this table and figure). Then, we calculated individual differential scores for HR, RMSSD, and SI by subtracting the values measured during the baseline phase from those measured during the subsequent phases (video, touch, and recovery) for each participant in each experimental block.

We used a model comparison approach specifying several predictors of interest for each dependent variable, and their statistical evidence was evaluated using Akaike Information Criterion (AIC; Akaike 1992) and AIC weights (Wagenmakers and Farrell 2004). Specifically, we compared a set of 14 mixed-effects models, including the fixed effects of experimental Phase (three-level factor: video, touch, and recovery), Block (two-level factor: babbling vs crying), Group (two-level factor: affective touch group vs non-affective touch group), and their interactions, see Table 1.

**Table 1.** Description of the models.

Model	Random and fixed factors included
Model 0 (null model)	Only random effect of participants: specified the hypothesis of no difference due to the independent variables and only accounted for individual variability
Model 1a	Random effect + fixed effect of Phase (video vs touch vs recovery)
Model 1b	Random effect + fixed effect of Block (babbling vs crying)
Model 1c	Random effect + fixed effect of Group (affective touch vs non-affective touch)
Model 2a	Random effect + additive Phase and Block effects
Model 2b	Random effect + additive Phase and Group effects
Model 2c	Random effect + additive Block and Group effects
Model 3a	Random effect + interaction effect between Phase and Block
Model 3b	Random effect + interaction effect between Phase and Group
Model 3c	Random effect + interaction effect between Block and Group
Model 4a	Random effect + interaction effect between Phase and Block with additive Group
Model 4b	Random effect + interaction effect between Phase and Group with additive Block
Model 4c	Random effect + interaction effect between Block and Group with additive Phase
Model 5	Random effect + three-way interaction effect between Phase, Block, and Group

Among the specified models, we selected the model that produces the lowest AIC value (Hooper et al. 2008) and quantified the strengths of evidence supporting this selection using its AIC weight, which can be interpreted as the conditional probability for each model of being the most plausible, given the data and the set of candidate models (Wagenmakers and Farrell 2004). Then we tested the effects predicted by the best model using analysis of deviance (ANOVA, Type III Wald chi-square test, ‘car’ package; Fox and Weisberg 2019) and *post-hoc* contrasts (‘emmeans’ package; Lenth 2024). Specifically, *post-hoc* contrasts were run on the phase factor, which includes three levels (video, touch, and recovery). Therefore, we performed three *t*-tests, and *P*-values were consequently adjusted for multiple comparisons using Bonferroni correction (*P* < .0167). Mixed-effect models were employed to account for the repeated-measure design of the experiment (i.e. blocks and phases nested within participants; Hooper et al. 2008). For analysing subjective and physiological variations, we used the ‘lmer’ function from the ‘lme4’ package (Bates et al. 2015), and *P* values were also calculated using the ‘lmerTest’ package (Kuznetsova et al. 2017). To assess the goodness of prediction, both conditional *R*^2^ (the proportion of variance explained by both fixed and random effects) and marginal *R*^2^ (the proportion of variance explained by fixed effects only, relative to total variance) were computed (Nakagawa and Schielzeth 2013). Elevated percentages of explained variance signify a more robust connection between the dependent variable and the predictors, indicating superior predictive performance of the model.

Finally, to explore the possible relationships between subjective affective states and physiological responses, we carried out correlational analyses using the ‘rcorr’ function from the Hmisc package, which returns both the Pearson’s correlation coefficient and the significance level of the association for all possible pairs of columns of a matrix.

## Results

### Subjective ratings: valence and arousal

**Figure 2.**
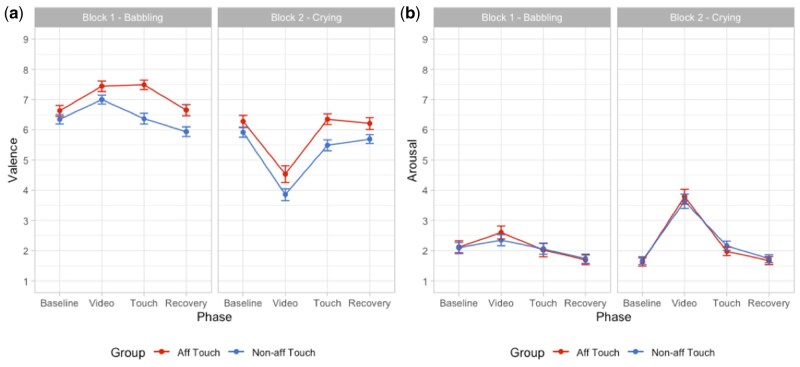
Panel (a) shows scores of valence (mean and standard error) after each experimental phase of each block. Panel (b) shows scores of arousal (mean and standard error) after each experimental phase of each block.

Figure 2 shows scores of valence and arousal to provide information about the overall evaluation of affective state during experimental phases. Please note that on the 1–9 SAM scale, valence ratings around 5 indicate neutrality (neither pleasantness nor unpleasantness), while scores below or above this value indicate unpleasantness and pleasantness, respectively (Lang et al. 2008).

To analyse the modulation of participants’ affective state in terms of valence and arousal, we compared a set of 14 mixed-effect models as described in the Statistical Analyses section.

#### Valence

**Figure 3.**
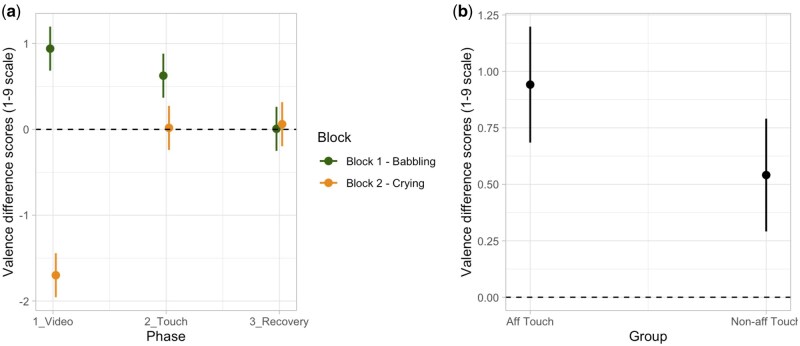
Plots of predicted values of valence modulation. Panel (a) shows the interaction effect between Phase and Block. Panel (b) shows the main effect of Group. Prediction lines and bars with confidence intervals (*n* participants = 92; *n* observations = 552). Dashed lines indicate the baseline level of valence.

For the modulation of valence, according to AIC and AIC weights, the best-fitting model was Model 4a (AIC = 1663.6, AIC weight = 0.790; *R*^2^ conditional = 0.456, *R*^2^ marginal = 0.380; for details on model comparison, see Table S2, see online supplementary material for a colour version of this table), which includes the interaction effect between Phase and Block with the additive effect of Group. The ANOVA on Model 4a revealed a main effect of Group (*χ*^2^[1, *N* = 92] = 11.35, *P <* .001) and an interaction between Phase and Block (*χ*^2^[2, *N* = 92] = 171.87, *P <* .001). *Post-hoc* contrasts revealed that participants showed a significant different modulation of valence between the first and the second block during the phases of video (babbling vs crying; *t* = 17.43, *P* < .001; *P*-value adjusted for multiple comparisons using Bonferroni correction, *P* < .0167), which persisted also during the touch phase (*t* = 4.02, *P* < .001; *P*-value adjusted for multiple comparisons using Bonferroni correction, *P* < .0167), but not during recovery (for details on the best model, ANOVA and *post-hoc* test, see Table S3 and Fig. S2, see online supplementary material for a colour version of this table and figure). These results indicate that participants reported an increase of valence when presented with the video of an infant babbling and a decrease when presented with a video of an infant crying. Moreover, participants receiving affective touch reported an overall greater increase in pleasantness compared to the group of participants receiving non-affective touch (Fig. 3).

#### Arousal

**Figure 4.**
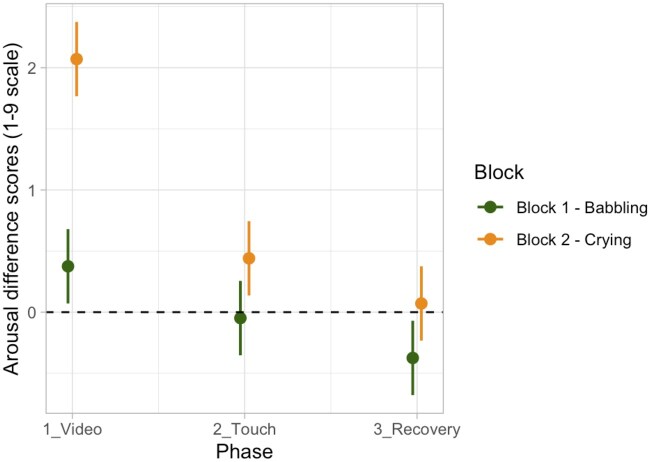
Plot of predicted values of arousal modulation, showing the interaction effect between Phase and Block. Prediction lines and bars with confidence intervals (*n* participants = 92; *n* observations = 552). Dashed lines indicate the baseline level of arousal.

For the modulation of arousal, according to AIC and AIC weights, the best-fitting model was Model 3a (AIC = 1707.1, ΔAIC = 175.08, AIC weight = 0.722, *R*^2^ conditional = 0.514, *R*^2^ marginal = 0.295; for details on model comparison, see Table S4, see online supplementary material for a colour version of this table), which includes the interaction effect between Phase and Block. The ANOVA on Model 3a revealed an interaction between Phase and Block (*χ*^2^ (2, *N* = 92) = 45.45, *P <* .001). *Post-hoc* contrasts revealed that participants showed a significant increase in arousal in all phases of the second block compared to the first block, in particular for the phase of video (babbling vs crying; *t* = −11.39, *P* < .001; *P*-value adjusted for multiple comparisons using Bonferroni correction, *P* < .0167). For details on the best Model, ANOVA, and *post-hoc* test, see Table S5 and Fig. S3 (see online supplementary material for a colour version of this table and figure). These results indicate that participants reported an increase in arousal when presented with the video of an infant crying (Fig. 4).

### Physiological measures: HR, RMSSD, and SI

#### Heart rate

**Figure 5.**
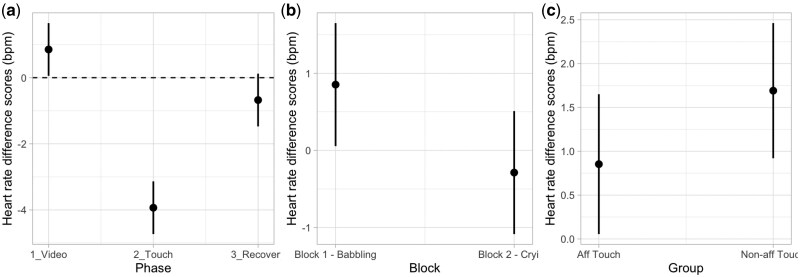
Plots of predicted values of heart rate (HR in bpm) modulation. Panel (a) shows the interaction effect of Phase. Panel (b) shows the effect of Block. Panel (c) shows the main effect of Group. Prediction lines and bars with confidence intervals (*n* participants = 92; *n* observations = 552). Dashed lines indicate the baseline level of HR.

To analyse the modulation of participants’ physiological responses, we compared a set of 14 mixed-effect models as described in the Statistical Analyses section. For the modulation of HR, according to AIC and AIC weights, the best-fitting model was Model 4a (AIC = 2841.6, ΔAIC = 161.65, AIC weight = 0.343, *R*^2^ conditional = 0.407, *R*^2^ marginal = 0.232; for details on model comparison, see Table S6, see online supplementary material for a colour version of this table), which includes the interaction effect between Phase and Block with the additive effect of Group. The ANOVA on Model 4a revealed main effects of Phase (*χ*^2^ (2, *N* = 92) = 131.93, *P <* .001), indicating an HR decrease during the tactile stimulation, and a main effect of Block (*χ*^2^ (1, *N* = 92) = 7.19, *P =* .007), indicating overall lower HR in the second (crying) compared to the first (babbling) experimental block (Fig. 5). Although not reaching the significance level, it could be interesting to note that a trend towards an interaction between Phase and Block (*χ*^2^ (2, *N* = 92) = 5.90, *P =* .052) emerged. Finally, a main effect of Group (*χ*^2^ = 4.18, *P =* .041) emerged, suggesting that the group of participants receiving affective touch showed, overall, lower HR compared to the group receiving non-affective touch (Fig. 5); for details on the best Model, ANOVA, and post-hoc test, see Table S7 and Fig. S4 (see online supplementary material for a colour version of this table and figure).

#### RMSSD index

**Figure 6.**
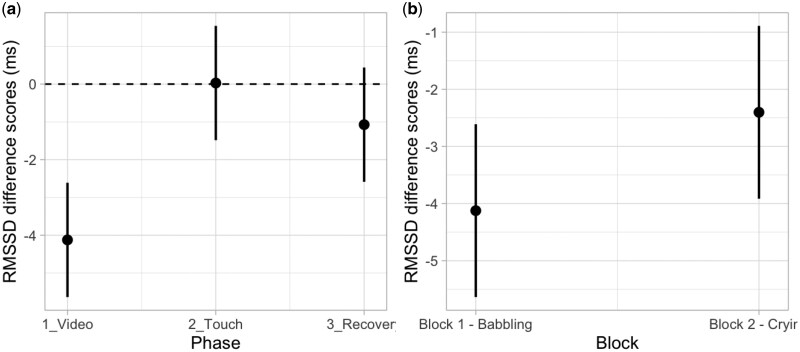
Plots of predicted values of heart rate variability (RMSSD index in ms) modulation. Panel (a) shows the main effect of Phase. Panel (b) shows the main effect of Block. Prediction lines and bars with confidence intervals (*n* participants = 92; *n* observations = 552). Dashed lines indicate the baseline level of RMSSD.

For the modulation of RMSSD, according to AIC and AIC weights, the best-fitting model was Model 2a (AIC = 3920.3, ΔAIC = 30.79, AIC weight = 0.298, *R*^2^ conditional = 0.229, *R*^2^ marginal = 0.049; for details on model comparison, see Table S8, see online supplementary material for a colour version of this table), which includes the additive effects of Phase and Block. The ANOVA on Model 2a revealed a main effect of Phase (*χ*^2^ (2, *N* = 92) = 28.09, *P <* .001). Specifically, *post-hoc* test suggests that the phase of video significantly differed from both the phase of touch (*t* = −5.11, *P* < .001; *P*-value adjusted for multiple comparisons using Bonferroni correction, *P* < .0167) and the phase of recovery (*t* = −3.05, *P* < .001; *P*-value adjusted for multiple comparisons using Bonferroni correction, *P* < .0167), indicating a decrease of HRV during video presentations and a return to baseline levels during the following phases (Fig. 6). A main effect of Block (*χ*^2^ = 6.75, *P=* .009) also emerged, suggesting overall lower HRV in the first (babbling) compared to the second (crying) experimental block (Fig. 6); for details on the best Model, ANOVA, and *post-hoc* test, see Table S9 and Fig. S5 (see online supplementary material for a colour version of this table and figure).

#### Stress index

**Figure 7.**
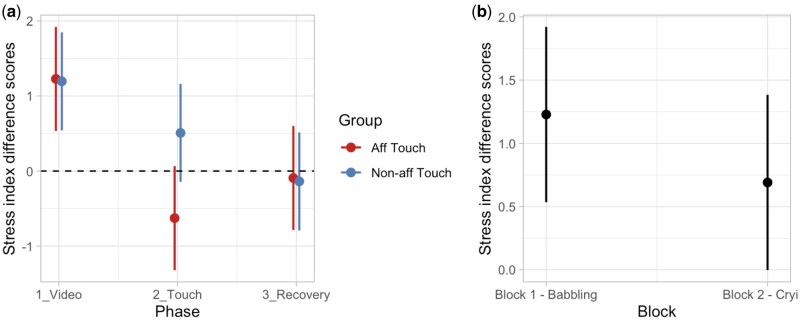
Plots of predicted values of stress index (SI) modulation. Panel (a) shows the interaction effect between Phase and Group. Panel (b) shows the main effect of Block. Prediction lines and bars with confidence intervals (*n* participants = 92; *n* observations = 552). Dashed lines indicate the baseline level of SI.

For the modulation of SI, according to AIC and AIC weights, the best-fitting model was Model 4b (AIC = 2677.8, ΔAIC = 6.28, AIC weight = 0.539, *R*^2^ conditional = 0.270, *R*^2^ marginal = 0.064; for details on model comparison, see Table S10, see online supplementary material for a colour version of this table), which includes the interaction effect between Phase and Group with the additive effect of Block. The ANOVA on Model 4b revealed a main effect of Block (*χ*^2^ (1, *N* = 92) = 6.41, *P =* .011) and an interaction between Phase and Group (*χ*^2^ (2, *N* = 92) = 6.78, *P =* .034). More specifically, the *post-hoc* test suggests that the two groups of participants showed a different modulation of SI during the tactile stimulation (*t* = −2.46, *P* = .015; *P*-value adjusted for multiple comparisons using Bonferroni correction, *P* < .0167); for details on the best Model, ANOVA, and *post-hoc* test, see Table S11 and Fig. S6 (see online supplementary material for a colour version of this table and figure). These results indicate participants showed an overall greater increase in SI during the first block (babbling) compared to the second block (crying). Importantly, when exposed to tactile stimulation after the video presentation, the group receiving affective touch showed a lower SI compared to the group receiving non-affective touch (Fig. 7).

### Correlations between subjective and physiological measures

**Figure 8.**
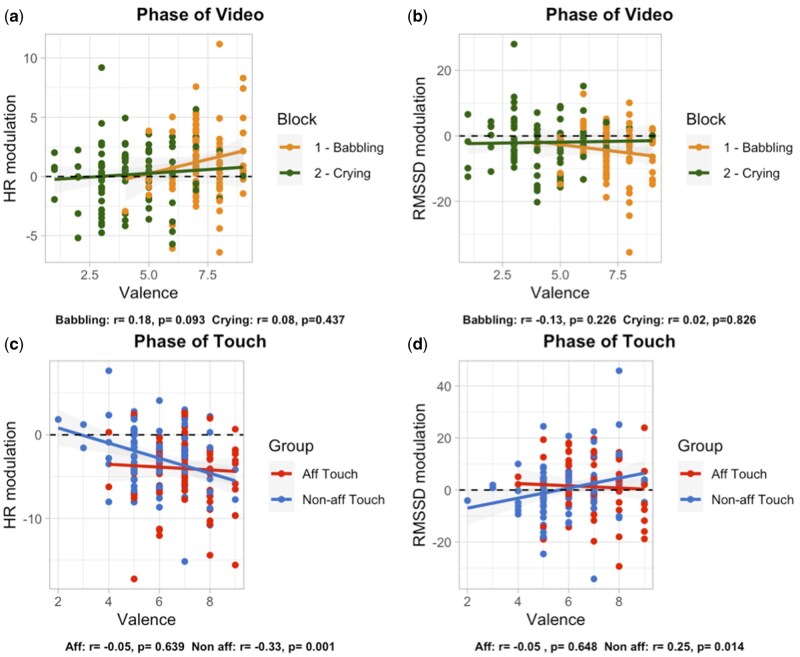
Plots of correlations between subjective scores of valence and heart rate modulation and RMSSD modulation during the phase of video presentation [Panels (a) and (b), respectively] and during the phase of touch [Panels (c) and (d)].

To investigate the association between subjective affective states and physiological measures during video presentation and tactile stimulation, we ran exploratory correlational analysis focusing on subjective measures (absolute values of valence and arousal on the 1–9 SAM scale) and modulation of physiological responses (differential scores of HR, RMSSD, and SI compared to baseline levels). Considering the phase of video, we observed a positive correlation between valence and HR modulation (*r* = 0.23, *P* = .002; *n* observations = 184) and a small negative correlation between valence and RMSSD modulation (*r* = −0.15, *P* = .045; *n* observations = 184). These correlations seem to be driven mainly by the responses in the first experimental block (Fig. 8a and b).

Considering the phase of touch, we observed a negative correlation between valence and HR modulation (*r* = −0.24, *P* = .001; *n* observations = 184) and a small positive correlation between valence and RMSSD (*r* = 0.12, *P* = .117; *n* observations = 184). More specifically, looking at the correlations in the two groups of participants receiving affective vs non-affective touch, results suggest that both correlations are stronger for the non-affective touch group (HR modulation: *r* = −0.33, *P* = .001; RMSSD modulation: *r* = 0.25, *P* = .014; *n* observations = 98; Fig. 8c and d).

No significant correlations emerged between subjective scores of arousal and physiological responses.

## Discussion

Our social environment is constantly overloaded with affective information reaching our senses through different sensory channels. Current theoretical models are focused on visual and auditory processes that convey critical emotional cues (e.g. facial emotional expressions). However, touch represents a powerful means of communication and regulation of emotions (Hertenstein et al. 2009). In particular, affective touch targeting CT afferents may represent a source of emotional support and comfort that scaffolds and promotes the development of self-regulatory mechanisms across the lifespan (Farroni et al. 2022, Fotopoulou et al. 2022). In the present study, we delved into subjective and physiological responses to emotionally salient videos (infant babbling vs infant crying) and how different tactile stimulations (affective vs non-affective touch) may modulate affective responses in young childless adults.

### Subjective responses during emotion-inducing videos and touch

First we analysed the subjective measures of valence and arousal to assess whether the experimental manipulations were effective in modulating subjective affective states. Results suggest that participants reported a significant increase in pleasantness after watching the video of an infant babbling. On the contrary, a negatively valenced affective state accompanied by increased arousal emerged after participants were presented with the video of an infant crying. These response patterns indicate that our video stimuli were effective in eliciting a subjective modulation of affective state in young adult participants. Moreover, the group of participants receiving affective touch reported a greater increase in pleasantness compared to the non-affective touch group, confirming that different tactile stimulations specifically elicited a modulation of subjective affective states, in line with previous findings consistently reporting an association between CT-optimal touch and pleasant subjective feelings (Löken et al. 2009, Cruciani et al. 2021, Crucianelli et al. 2022).

### Physiological responses during video presentation

Beyond subjective reports, we employed physiological measures to unveil underlying autonomic mechanisms that may support emotional processing and regulation. Specifically, we considered three indices extracted from the IBI series of the ECG, which are related to different cardiac dynamics: HR, as an index of changes in cardiac rhythm controlled by both the sympathetic and parasympathetic branches of the ANS; RMSSD, as an index of vagally mediated HR variability linked to self-regulatory mechanisms; and SI, as an index of sympathetic activation during arousing or stressful events.

Considering the phase of video presentation, we expected a stronger physiological response during the crying scene compared to the babbling scene. However, results indicated (i) a more pronounced HR increase during the babbling compared to the crying scene, (ii) a decrease in vagally mediated HRV (RMSSD index) during the video presentation and overall lower HRV in the first block, including the video of an infant babbling, compared to the second block, including the video of an infant crying, and finally (iii) an overall increase in sympathetic activity (SI) during the video presentation and higher SI in the first (babbling) compared to the second (crying) experimental block. This response pattern suggests both activation of the sympathetic system and vagal withdrawal during the presentation of videos with emotional content, in line with previous research indicating an increase in HR in response to emotional film clips inducing different emotions (Fernández et al. 2012, Fernández-Aguilar et al. 2020), as well as a decrease in HRV in response to emotional stimuli, regardless of valence (Balzarotti et al. 2017). Unlike our expectations, these responses seemed to be more pronounced during the positive (babbling) compared to the negative and more arousing video (crying), as also supported by the significant correlations between cardiac modulations and the valence ratings reported immediately after the video. Indeed, we found that more positive valence scores were related to both increases in HR and decreases in HRV, indicating autonomic activation during emotional processing. Although our correlational analyses are explorative and we invite caution in interpretation, it can be noted that this result is in line with previous evidence suggesting that amusement experience induced by an emotional film was related to cardiac dynamics, while the sadness experience was not (Mauss et al. 2005). Taken together, we interpret the physiological responses during video presentation as a marker of emotional engagement, supported by sympathetic activation and vagal withdrawal at the sinoatrial node, with no specific pattern indicative of emotional distress observed during the crying scene. It is important to note that reported arousal levels were generally low (see Table 1), although they increased after watching the infant crying, suggesting that the videos elicited modulation of affective states at moderate intensity levels. Future investigations should aim to induce stronger affective responses and emotional distress by employing highly arousing videos or first-person experiences (e.g. social exclusion paradigms).

### The buffering effect of touch

Considering the phase of touch, the results suggest a prominent cardiac deceleration, supported by an increase in vagally mediated activity (RMSSD index) and a decrease in sympathetic activity (SI index). Importantly, the specific type of touch that participants received (affective vs non-affective touch) modulated these autonomic responses. Indeed, the decrease in HR was larger for the group of participants experiencing affective touch compared to those experiencing non-affective touch, in line with previous studies consistently reporting HR deceleration during affective touch stimulation (Fairhurst et al. 2014; Pawling et al. 2017b). Moreover, affective touch compared to non-affective touch was more effective in reducing sympathetic activation (decreased SI index) after the emotional video. Notably, exploratory correlational analyses seem to suggest a link between HR/HRV modulations and subjective ratings of valence during non-affective touch. This result is intriguing, shedding light on individual differences that can modulate both subjective emotional experience and physiological responses to tactile stimulation. Specifically, our results may indicate that participants who reported a highly pleasant affective state following non-affective touch also showed a physiological modulation similar to those receiving the affective tactile stimulation (i.e. HR decrease and RMSSD increase). On the contrary, participants reporting neutral or negative valence did not show this cardiac response pattern. It is possible that, as suggested by Walker et al. (2022a, 2022b), the cardiac deceleration response during tactile stimulation denotes orienting to pleasantness rather than reflecting a CT-specific effect. This interpretation is in line with previous studies suggesting that not only bottom-up activation of the CT system but also subjective positive evaluation of touch may contribute to modulation of physiological and affective responses. For example, Pawling et al. (2017a, 2017b) found that caress-like tactile stimulations delivered on the CT-innervated forearm and on the non-CT-innervated palm were both evaluated as pleasant and elicited an HR decrease. Further investigation with larger inferential studies is needed to confirm our explorative finding and to shed light on the relationship between autonomic responses, affective touch, and individual differences.

Taken together, our physiological measures provided complementary information to better understand the complex autonomic mechanisms involved in regulating the affective states. It is worth pointing out that subjective measures confirmed the efficacy of our video stimuli in eliciting different emotional responses (i.e. a decrease in valence and an increase in arousal during the crying scene). However, at the physiological level, participants did not show stress-related responses (i.e. increase in HR and SI and a decreased in HRV during the crying scene), thus limiting the exploration and interpretation of the buffering effect of touch in the context of emotional distress. Nevertheless, our results highlight the specific responses to tactile stimulation that strongly suggest a reduction of sympathetic activity associated with vagal dominance. Thus, our results are in line with the well-established hypothesis of the CT system having a specific role in signalling the social affective relevance of touch by activating appetitive motivational reflexes that function to increase attention and facilitate perceptual processing (Morrison et al. 2010, Walker et al. 2022a, 2022b). In this perspective, affective touch represents a crucial element of self-regulatory mechanisms with the potential of rebalancing internal homeostatic needs in a given socio-emotional context and modulating behavioural responses to concurrently occurring events.

### Limitations and future perspectives

It is worth mentioning that the present study has some limitations. First, we used a fixed block experimental design in which all participants were first presented with the infant babbling video and then with the infant crying video. This methodological choice was made to prevent possible long-lasting effects of the crying scene. However, this might have affected our results, in particular at the physiological level. It is possible that the repetitive design of the experiment (baseline-video-touch-recovery) and the relatively long duration of each phase (2 min) induced habituation, leading participants to show more blunted responses in the second compared to the first experimental block.

Second, a female experimenter performed tactile stimulation on all participants, both males and females. This might have resulted in a possible gender effect involving top-down processes related to who was performing the touch. To reduce this potential bias, we decided to use an object-mediated stimulation (brushing), which has been shown to optimally activate CT afferents and reliably evoke pleasant feelings (e.g. Löken et al. 2009), as typically used in studies of affective touch (for a review, see Cruciani et al. 2021). Another important point is that in our study the majority of participants were female (80 females and 12 males), thus preventing the possibility to specifically address gender-related differences. Future studies should include a more balanced sample and specifically investigate whether females and males respond differently to emotional videos of infant crying and to subsequent tactile stimuli. Moreover, it would also be desirable to involve two experimenters of different genders in order to ensure gender congruency between participants and experimenters.

Third, individual variability deserves some attention. Physiological measures are strongly affected by individual characteristics. As shown by the baseline values (Fig. 4), we found large inter-individual variability. This suggests the importance of considering differential scores, which account for different individual baseline levels of cardiac activity, and of using mixed models including the random effect of participants. Still, inter-individual contributions should be further investigated in future studies using larger samples and, possibly, more individualized and longitudinal assessments. In experimental psychology, implicit generalizations are frequently made: not only from experimental samples to the broader population but also from group-level effects, assuming that these phenomena are stable and present in every individual (Roth et al. 2024). However, not all participants exhibit a replicable and consistent group-level effect at the individual level. This perspective leads to a paradigm shift in which individual differences are no longer viewed as random data errors but as an essential source of information.

Fourth, the ecological validity of the study should be considered in terms of both stress-related stimuli and tactile interactions. To elicit emotional responses, we used video stimuli, which were effective at the subjective level but did not elicit physiological stress-related responses during the crying scene. Previous research with more structured stress paradigms (i.e. Trier Social Stress Test; Ditzen et al. 2007) or naturally occurring stress events (i.e. Covid-19 pandemic; Schneider et al. 2023) provided initial evidence of the beneficial effects of tactile interactions in regulating physiological and hormonal responses in prolonged and ecologically valid contexts. Additionally, receiving touch from a significant person (i.e. parent, spouse) may play a crucial role in offering comforting support and promoting well-being (Ditzen et al. 2007, Püschel et al. 2022). Future investigations should investigate the preventive effects of affective touch in reducing emotional distress when provided prior to the emotional and stress-related stimulus, possibly in ecologically valid and meaningful social contexts.

A final consideration concerns the lack of neural measures that might provide a more comprehensive understanding of the role of affective touch in modulating emotional changes. Future investigations should include both cardiac and neural measures in order to specifically explore the integration of physiological inputs in the brain during emotional processing. Recent studies have started to uncover the complexity of brain–heart coupling, focusing on the bidirectional interplay between ascending inputs from the heart and central neural processing involved in key aspects of emotional experience (Candia-Rivera et al. 2022, Banik et al. 2024, Cai et al. 2024). Specifically, cardiac parasympathetic activity, reflected by modulations in HRV, appears to play a causal role in the processing of emotional arousal, sustaining both ascending and descending brain–heart interactions (Candia-Rivera et al. 2022): vagally mediated HRV changes support electroencephalographical oscillations, particularly in the theta band, resulting in a heart–brain coupling (heart-to-brain interplay). In turn, modulation of theta band oscillations triggers cortical activation and top-down neural control of the heart (brain-to-heart interplay). Moreover, initial evidence suggests the brain–heart coupling in the alpha band can differentiate between different levels of emotional valence (Banik et al. 2024). These promising results pave the way for further investigations focusing on brain–heart interplay to uncover possible mechanisms mediating the effects of affective touch in emotional processing and autonomic self-regulation in more complex and ecological multisensory scenarios.

## Conclusions

In conclusion, emotional processes occur in a complex interaction between the social environment, the brain, and the organism. The present study works towards unpacking subjective and physiological components of emotional processing, focusing on the role of affective touch in facilitating regulatory mechanisms after exposure to emotionally salient videos. Our results suggest that affective touch increased the positive valence of subjective affective state and elicited larger HR decreases compared to non-affective touch. Moreover, affective touch was effective in restoring HRV to baseline levels and in reducing the SI after emotional activation in response to the videos. These findings provide evidence in support of the effect of affective touch in decreasing sympathetic activity and increasing vagal dominance, which may play an important role in autonomic self-regulation. Moreover, our results underline the importance of integrating different physiological and subjective measures to better understand the complex mechanisms supporting emotional processing and regulation.

## Supplementary Material

nsaf090_Supplementary_Data

## Data Availability

The data and scripts for statistical analyses underlying this article are available at https://osf.io/ujvng/? view_only=5df368396e564f20904124e34a50fa32

## References

[nsaf090-B1] Ackerley R , Backlund Wasling H, Liljencrantz J et al Human C-tactile afferents are tuned to the temperature of a skin-stroking caress. J Neurosci 2014;34:2879–83. 10.1523/JNEUROSCI.2847-13.2014 24553929 PMC3931502

[nsaf090-B2] Akaike H. Information theory and an extension of the maximum likelihood principle. In: Kotz S, Johnson NL (eds), Breakthroughs in Statistics: Foundations and Basic Theory. New York, NY: Springer, 1992, 610–24. 10.1007/978-1-4612-0919-5_38

[nsaf090-B3] Anbalagan T , Nath MK, Vijayalakshmi D et al Analysis of various techniques for ECG signal in healthcare, past, present, and future. Biomed Eng Adv 2023;6:100089.

[nsaf090-B4] Babo-Rebelo M , Richter CG, Tallon-Baudry C. Neural responses to heartbeats in the default network encode the self in spontaneous thoughts. J Neurosci 2016;36:7829–40. 10.1523/JNEUROSCI.0262-16.2016 27466329 PMC4961773

[nsaf090-B5] Balzarotti S , Biassoni F, Colombo B et al Cardiac vagal control as a marker of emotion regulation in healthy adults: a review. Biol Psychol 2017;130:54–66. 29079304 10.1016/j.biopsycho.2017.10.008

[nsaf090-B6] Bartlett E , McMahon C. The cognitive, affective and physiological impact of infant crying: a comparison of two laboratory methodologies. J Reprod Infant Psychol 2016;34:196–209. 10.1080/02646838.2015.1113515

[nsaf090-B7] Bates D , Mächler M, Bolker B et al Fitting linear mixed-effects models using lme4. J Stat Soft 2015;67:1–48. 10.18637/jss.v067.i01

[nsaf090-B9] Baevsky RM , Chernikova AG; Institute of Biomedical Problems of the Russian Academy of Sciences. Heart rate variability analysis: physiological foundations and main methods. Cardiometry 2017;10:66–76.

[nsaf090-B10] Banik S , Kumar H, Ganapathy N et al Assessment of emotion elicitation using multimodal physiological sensors and phase synchronization. IEEE Sens Lett 2024;8:1–4. 10.1109/LSENS.2024.3426562

[nsaf090-B11] Boehme R , Hauser S, Gerling GJ et al Distinction of self-produced touch and social touch at cortical and spinal cord levels. Proc Natl Acad Sci U S A 2019;116:2290–9. 10.1073/pnas.1816278116 30670645 PMC6369791

[nsaf090-B12] Bradley MM , Lang PJ. Measuring emotion: the self-assessment manikin and the semantic differential. J Behav Ther Exp Psychiatry 1994;25:49–59. 10.1016/0005-7916(94)90063-9 7962581

[nsaf090-B13] Brisinda D , Picerni M, Fenici P et al Synthetic assessment of cardiac autonomic modulation and Baevsky stress index in patients with synucleinopathies. Eur Heart J 2024;45:ehae666.3017.

[nsaf090-B14] Cai Z , Gao H, Wu M et al Physiologic network-based brain-heart interaction quantification during visual emotional elicitation. IEEE Trans Neural Syst Rehabil Eng 2024;32:2482–91. 10.1109/TNSRE.2024.3424543. 38976471

[nsaf090-B15] Candia-Rivera D , Boehme R, Salamone PC. Autonomic modulations to cardiac dynamics in response to affective touch: Differences between social touch and self-touch. *IEEE Trans Affective Comput* 2025;16:1996–2005. 10.1109/TAFFC.2025.3548778

[nsaf090-B16] Candia-Rivera D , Catrambone V, Thayer JF et al Cardiac sympathetic-vagal activity initiates a functional brain–body response to emotional arousal. Proc Natl Acad Sci U S A 2022;119:e2119599119. 10.1073/pnas.2119599119 35588453 PMC9173754

[nsaf090-B17] Candia-Rivera D , de Vico Fallani F, Boehme R et al Linking heartbeats with the cortical network dynamics involved in self-social touch distinction. Commun Biol 2025;8:52. 10.1038/s42003-024-07448-z 39809818 PMC11733256

[nsaf090-B18] Cascio CJ , Moore D, McGlone F. Social touch and human development. Dev Cogn Neurosci 2019;35:5–11. 29731417 10.1016/j.dcn.2018.04.009 PMC6968965

[nsaf090-B19] Carozza S , Leong V. The role of affectionate caregiver touch in early neurodevelopment and parent–infant interactional synchrony. Front Neurosci 2021;14:613378. 10.3389/fnins.2020.613378 33584178 PMC7873991

[nsaf090-B20] Chu B , Marwaha K, , Sanvictores T, et al Physiology, stress reaction. In: StatPearls. StatPearls Publishing, Treasure Island (FL). 2024. https://www.ncbi.nlm.nih.gov/books/NBK541120/2024 31082164

[nsaf090-B21] Coan JA , Schaefer HS, Davidson RJ. Lending a hand: social regulation of the neural response to threat. Psychol Sci 2006;17:1032–9. 10.1111/j.1467-9280.2006.01832.x 17201784

[nsaf090-B22] Cohen-Bendahan CCC , van Doornen LJP, de Weerth C. Young adults’ reactions to infant crying. Infant Behav Dev 2014;37:33–43. 10.1016/j.infbeh.2013.12.004 24463036

[nsaf090-B23] Crucianelli L , Chancel M, Ehrsson HH. Modeling affective touch pleasantness across skin types at the individual level reveals a reliable and stable basic function. J Neurophysiol 2022;128:1435–52. 10.1152/jn.00179.2022 36260710

[nsaf090-B24] Cruciani G , Zanini L, Russo V et al Pleasantness ratings in response to affective touch across hairy and glabrous skin: a meta-analysis. Neurosci Biobehav Rev 2021;131:88–95. 10.1016/j.neubiorev.2021.09.026 34537264

[nsaf090-B25] Deuchars SA , Lall VK. Sympathetic preganglionic neurons: properties and inputs. Compr Physiol 2015;5:829–69. 10.1002/cphy.c140020 25880515

[nsaf090-B26] Ditzen B , Neumann ID, Bodenmann G et al Effects of different kinds of couple interaction on cortisol and heart rate responses to stress in women. Psychoneuroendocrinology 2007;32:565–74. 10.1016/j.psyneuen.2007.03.011 17499441

[nsaf090-B27] Dondi M , Simion F, Caltran G. Can newborns discriminate between their own cry and the cry of another newborn infant? Dev Psychol 1999;35:418–26. 10.1037//0012-1649.35.2.418 10082012

[nsaf090-B28] Draghici AE , Taylor JA. The physiological basis and measurement of heart rate variability in humans. J Physiol Anthropol 2016;35:22. 10.1186/s40101-016-0113-7 27680542 PMC5039876

[nsaf090-B29] Faig KE , Smith KE, Dimitroff SJ. Somatovisceral influences on emotional development. Emot Rev 2023;15:127–44. 10.1177/17540739231163180 38148757 PMC10751027

[nsaf090-B30] Fairhurst MT , Löken L, Grossmann T. Physiological and behavioral responses reveal 9-month-old infants’ sensitivity to pleasant touch. Psychol Sci 2014;25:1124–31. 10.1177/0956797614527114 24681587 PMC4017181

[nsaf090-B31] Farroni T , Della Longa L, Valori I. The self-regulatory affective touch: a speculative framework for the development of executive functioning. Curr Opin Behav Sci 2022;43:167–73. 10.1016/j.cobeha.2021.10.007

[nsaf090-B32] Fernández C , Pascual JC, Soler J et al Physiological responses induced by emotion-eliciting films. Appl Psychophysiol Biofeedback 2012;37:73–9. 10.1007/s10484-012-9180-7 22311202

[nsaf090-B33] Fernández-Aguilar L , Latorre JM, Martínez-Rodrigo A et al Differences between young and older adults in physiological and subjective responses to emotion induction using films. Sci Rep 2020;10:14548. 10.1038/s41598-020-71430-y 32883988 PMC7471684

[nsaf090-B34] Field T. Touch for socioemotional and physical well-being: a review. Dev Rev 2010;30:367–83.

[nsaf090-B35] Fotopoulou A , von Mohr M, Krahé C. Affective regulation through touch: homeostatic and allostatic mechanisms. Curr Opin Behav Sci 2022;43:80–7. 10.1016/j.cobeha.2021.08.008 34841013 PMC7612031

[nsaf090-B201] Fox J , Weisberg S. An R Companion to Applied Regression, Third edition. Sage, Thousand Oaks CA, 2019. https://www.john-fox.ca/Companion/

[nsaf090-B36] Gemignani M , Giannotti M, Rigo P et al Attentional bias to infant faces might be associated with previous care experiences and involvement in childcare in same-sex mothers. Int J Clin Health Psychol 2024;24:100419. 10.1016/j.ijchp.2023.100419 37885912 PMC10598538

[nsaf090-B37] Gordon I , Voos AC, Bennett RH et al Brain mechanisms for processing affective touch. Hum Brain Mapp 2013;34:914–22. 10.1002/hbm.21480 22125232 PMC6869848

[nsaf090-B38] Hechler C , Beijers R, de Weerth C. Young adults’ reactions to infant crying. Infant Behavior & Development 2015;38:41–8. 10.1016/j.infbeh.2014.12.006 25597613

[nsaf090-B39] Heiss S , Vaschillo B, Vaschillo EG et al Heart rate variability as a biobehavioral marker of diverse psychopathologies: a review and argument for an ‘ideal range’. Neurosci Biobehav Rev 2021;121:144–55. 10.1016/j.neubiorev.2020.12.004 33309905

[nsaf090-B40] Hertenstein MJ , Holmes R, McCullough M et al The communication of emotion via touch. Emotion 2009;9:566–73. 10.1037/a0016108 19653781

[nsaf090-B41] Holt-Lunstad J , Birmingham WA, Light KC. Influence of a ‘warm touch’ support enhancement intervention among married couples on ambulatory blood pressure, oxytocin, alpha amylase, and cortisol. Psychosom Med 2008;70:976–85. 10.1097/PSY.0b013e318187aef7 18842740

[nsaf090-B42] Hooper D , Coughlan J, Mullen M. Structural equation modelling: guidelines for determining model fit. *Electron J Bus Res Methods* 2008;6:53–60. 10.21427/D7CF7R

[nsaf090-B43] Huizinga JD , Chen JH, Hussain A et al Determining autonomic sympathetic tone and reactivity using Baevsky’s stress index. Am J Physiol Regul Integr Comp Physiol 2025;328:R562–77. 40013894 10.1152/ajpregu.00243.2024

[nsaf090-B44] Kidd T , Devine SL, Walker SC. Affective touch and regulation of stress responses. Health Psychol Rev 2023;17:60–77. 10.1080/17437199.2022.2143854 36346350

[nsaf090-B45] Kirsch LP , Besharati S, Papadaki C et al Damage to the right insula disrupts the perception of affective touch. eLife 2020;9:e47895. 10.7554/eLife.47895 31975686 PMC7043887

[nsaf090-B03798337] Kissin I , McDanal J, Brown PT et al Sympathetic blockade increases tactile sensitivity. Anesth Analg 1987;66:1251–5. 3688497

[nsaf090-B46] Kuznetsova A , Brockhoff PB, Christensen RHB. lmerTest package: tests in linear mixed effects models. J Stat Soft 2017;82:1–26. 10.18637/jss.v082.i13

[nsaf090-B47] Lang PJ , Bradley MM. Emotion and the motivational brain. Biol Psychol 2010;84:437–50. 10.1016/j.biopsycho.2009.10.007 19879918 PMC3612949

[nsaf090-B48] Lang PJ , Bradley MM, Cuthbert BN. International affective picture system (IAPS): affective ratings of pictures and instruction manual. Technical report A-7. University of Florida, Gainesville, FL, 2008.

[nsaf090-B49] Lenth R. emmeans: estimated marginal means, aka least-squares means. R package version 1.10.3, https://rvlenth.github.io/emmeans/. 2024.

[nsaf090-B50] Löken LS , Wessberg J, Morrison I et al Coding of pleasant touch by unmyelinated afferents in humans. Nat Neurosci 2009;12:547–8. 10.1038/nn.2312 19363489

[nsaf090-B51] Mauss IB , Levenson RW, McCarter L et al The tie that binds? Coherence among emotion experience, behavior, and physiology. Emotion 2005;5:175–90. 10.1037/1528-3542.5.2.175 15982083

[nsaf090-B72049462] McCraty R , Shaffer F. Heart rate variability: New perspectives on physiological mechanisms, assessment of self-regulatory capacity, and health risk. Glob Adv Health Med 2015;4:46–61. 10.7453/gahmj.2014.073 PMC4311559 25694852

[nsaf090-B52] Meijer LL , Ruis C, van der Smagt MJ et al Neural basis of affective touch and pain: a novel model suggests possible targets for pain amelioration. J Neuropsychol 2022;16:38–53. 10.1111/jnp.12250 33979481 PMC9290016

[nsaf090-B53] Melo HM , Martins TC, Nascimento LM et al Ultra‐short heart rate variability recording reliability: the effect of controlled paced breathing. . *Ann Noninvasive Electrocardiol* 2018;*23*:e12565. 10.1111/anec.12565 PMC6931441 29863781

[nsaf090-B55] McGlone F , Wessberg J, Olausson H. Discriminative and affective touch: sensing and feeling. Neuron 2014;82:737–55. 10.1016/j.neuron.2014.05.001 24853935

[nsaf090-B56] Morrison I. ALE meta-analysis reveals dissociable networks for affective and discriminative aspects of touch. Hum Brain Mapp 2016a;37:1308–20. 10.1002/hbm.23103 26873519 PMC5066805

[nsaf090-B57] Morrison I. Keep calm and cuddle on: social touch as a stress buffer. Adapt Human Behav Physiol 2016b;2:344–62. 10.1007/s40750-016-0052-x

[nsaf090-B58] Morrison I , Löken LS, Olausson H. The skin as a social organ. Exp Brain Res 2010;204:305–14. 10.1007/s00221-009-2007-y 19771420

[nsaf090-B59] Munoz ML , Roon A, van Riese H et al Validity of (ultra-)short recordings for heart rate variability measurements. PLoS One 2015;10:e0138921. 10.1371/journal.pone.0138921 26414314 PMC4586373

[nsaf090-B202] Nakagawa S , Schielzeth H A general and simple method for obtaining R2 from generalized linear mixed-effects models. Methods Ecol Evol 2013;4:133–42. 10.1111/j.2041-210x.2012.00261.x

[nsaf090-B60] Norman GJ , Berntson GG, Cacioppo JT. Emotion, somatovisceral afference, and autonomic regulation. Emot Rev 2014;6:113–23. 10.1177/1754073913512006

[nsaf090-B61] Nunan D , Sandercock GRH, Brodie DA. A quantitative systematic review of normal values for short-term heart rate variability in healthy adults. Pacing Clin Electrophysiol 2010;33:1407–17. 10.1111/j.1540-8159.2010.02841.x 20663071

[nsaf090-B62] Nussinovitch U , Elishkevitz KP, Katz K et al Reliability of ultra‐short ECG indices for heart rate variability. Ann Noninvasive Electrocardiol 2011;16:117–22. 21496161 10.1111/j.1542-474X.2011.00417.x PMC6932379

[nsaf090-B63] Ognev AS , Zernov VA, Likhacheva EV et al; Validity of cardiometric performance data: an integral part of complex assessment of training session effectiveness. Cardiometry 2019;14:96–100.

[nsaf090-B64] Olausson H , Wessberg J, Morrison I et al The neurophysiology of unmyelinated tactile afferents. Neurosci Biobehav Rev 2010;34:185–91. 10.1016/j.neubiorev.2008.09.011 18952123

[nsaf090-B65] Packheiser J , Hartmann H, Fredriksen K et al A systematic review and multivariate meta-analysis of the physical and mental health benefits of touch interventions. Nat Hum Behav 2024;8:1088–107. 10.1038/s41562-024-01841-8 38589702 PMC11199149

[nsaf090-B66] Pan J , Tompkins WJ. A real-time QRS detection algorithm. IEEE Trans Biomed Eng 1985;32:230–6. 10.1109/TBME.1985.325532 3997178

[nsaf090-B67] Pawling R , Cannon PR, McGlone FP et al C-tactile afferent stimulating touch carries a positive affective value. PLoS One 2017a;12:e0173457. 10.1371/journal.pone.0173457 28282451 PMC5345811

[nsaf090-B68] Pawling R , Trotter P, Mcglone F et al A positive touch: C-tactile afferent targeted skin stimulation carries an appetitive motivational value. Biol Psychol 2017b;129:186–94. 10.1016/j.biopsycho.2017.08.057 28865933

[nsaf090-B69] Pham T , Lau ZJ, Chen SHA et al Heart rate variability in psychology: a review of HRV indices and an analysis tutorial. Sensors (Basel) 2021;21:3998. 10.3390/s21123998 34207927 PMC8230044

[nsaf090-B70] Püschel I , Reichert J, Friedrich Y et al Gentle as a mother’s touch: C-tactile touch promotes autonomic regulation in preterm infants. Physiol Behav 2022;257:113991. 10.1016/j.physbeh.2022.113991 36242858

[nsaf090-B3261186] R Core Team (2023). R: A Language and Environment for Statistical Computing. R Foundation for Statistical Computing, Vienna, Austria. https://www.R-project.org

[nsaf090-B203] Roth L , Jordan V, Schwarz S et al Don’t SNARC me now! Intraindividual variability of cognitive phenomena—Insights from the Ironman paradigm. Cognition 2024;248:105781. 10.1016/j.cognition.2024.105781 38663115

[nsaf090-B71] Ruffman T , Then R, Cheng C et al Lifespan differences in emotional contagion while watching emotion-eliciting videos. PLoS One 2019;14:e0209253. 10.1371/journal.pone.0209253 30657754 PMC6338362

[nsaf090-B72] Sahoo TK , Mahapatra A, Ruban N. Stress index calculation and analysis based on heart rate variability of ECG signal with arrhythmia. In: 2019 Innovations in Power and Advanced Computing Technologies (i-PACT). IEEE, Vol. 1, 2019, 1–7. 10.1109/i-PACT44901.2019.8959524

[nsaf090-B73] Sailer U , Triscoli C, Häggblad G et al Temporal dynamics of brain activation during 40 minutes of pleasant touch. Neuroimage 2016;139:360–7. 10.1016/j.neuroimage.2016.06.031 27338514

[nsaf090-B74] Schneider E , Hopf D, Aguilar-Raab C et al Affectionate touch and diurnal oxytocin levels: an ecological momentary assessment study. Elife 2023;12:e81241. 10.7554/eLife.81241 37252874 PMC10229112

[nsaf090-B75] Shaffer F , Ginsberg JP. An overview of heart rate variability metrics and norms. Front Public Health 2017;5:258. 10.3389/fpubh.2017.00258 29034226 PMC5624990

[nsaf090-B76] Stern RM , William JR, Karen SQ. Psychophysiological Recording, 2nd edn. New York: Oxford Academic (online edn, 22 Mar. 2012), 2000. 10.1093/acprof:oso/9780195113594.001.0001

[nsaf090-B77] Thayer JF , Brosschot JF. Psychosomatics and psychopathology: looking up and down from the brain. Psychoneuroendocrinology 2005;30:1050–8. 10.1016/j.psyneuen.2005.04.014 16005156

[nsaf090-B78] Thayer JF , Lane RD. A model of neurovisceral integration in emotion regulation and dysregulation. J Affect Disord 2000;61:201–16. 10.1016/s0165-0327(00)00338-4 11163422

[nsaf090-B79] Thayer JF , Lane RD. Claude Bernard and the heart-brain connection: further elaboration of a model of neurovisceral integration. Neurosci Biobehav Rev 2009;33:81–8. 10.1016/j.neubiorev.2008.08.004 18771686

[nsaf090-B80] Thayer JF , Siegle GJ. Neurovisceral integration in cardiac and emotional regulation. IEEE Eng Med Biol Mag 2002;21:24–9. 10.1109/memb.2002.1032635 12222113

[nsaf090-B81] Thompson-Booth C , Viding E, Mayes LC et al Here’s looking at you, kid: attention to infant emotional faces in mothers and non-mothers. Dev Sci 2014;17:35–46. 10.1111/desc.12090 24341972 PMC4352331

[nsaf090-B82] Triscoli C , Croy I, Steudte-Schmiedgen S et al Heart rate variability is enhanced by long-lasting pleasant touch at CT-optimized velocity. Biol Psychol 2017;128:71–81. 10.1016/j.biopsycho.2017.07.007 28723347

[nsaf090-B83] Vallbo ÅB , Olausson H, Wessberg J. Unmyelinated afferents constitute a second system coding tactile stimuli of the human hairy skin. J Neurophysiol 1999;81:2753–63. 10368395 10.1152/jn.1999.81.6.2753

[nsaf090-B84] Van Puyvelde M , Gorissen A-S, Pattyn N et al Does touch matter? The impact of stroking versus non-stroking maternal touch on cardio-respiratory processes in mothers and infants. Physiol Behav 2019;207:55–63. 10.1016/j.physbeh.2019.04.024 31047950

[nsaf090-B86] Wagenmakers E-J , Farrell S. AIC model selection using Akaike weights. Psychon Bull Rev 2004;11:192–6. 10.3758/BF03206482 15117008

[nsaf090-B87] Walker SC , Marshall A, Pawling R. Psychophysiology and motivated emotion: testing the affective touch hypothesis of C-tactile afferent function. Curr Opin Behav Sci 2022a;43:131–7. 10.1016/j.cobeha.2021.10.004

[nsaf090-B88] Walker SC , Cavieres A, Peñaloza-Sancho V et al C-low threshold mechanoafferent targeted dynamic touch modulates stress resilience in rats exposed to chronic mild stress. Eur J Neurosci 2022b;55:2925–38. 10.1111/ejn.14951 32852872

[nsaf090-B89] Wu Y , Gu R, Yang Q et al How do amusement, anger and fear influence heart rate and heart rate variability? Front Neurosci 2019;13:1131. 10.3389/fnins.2019.01131 31680848 PMC6813458

