# Supplementary material for: Does affective touch buffer emotional distress? Insights from subjective and physiological indices
Source: Soc Cogn Affect Neurosci. 2025 Sep 5;20(1):nsaf090. doi: 10.1093/scan/nsaf090 (PMC12478029; doi:10.1093/scan/nsaf090)
Supplement: nsaf090_Supplementary_Data [file nsaf090_supplementary_data.docx]

**SUPPLEMENTARY MATERIALS**

Does affective touch buffer emotional distress? Insights from subjective and physiological indices

*Della Longa Letizia, Sarlo Michela, Farroni Teresa*

1. **Preliminary analyses on baseline physiological indices**

We controlled for potential confounding differences in individual physiological activity by running preliminary analysis on baseline levels of all cardiac indices of interest: heart rate (HR), heart rate variability (RMSSD index) and stress index (SI).

Table S1 shows descriptive statistics and analysis, considering baseline levels of our physiological variables of interest. For each dependent variable and we performed a 2×2 ANOVA including the effect of Block (babbling vs crying), Group (affective vs non-affective touch) and their interaction. No significant effects of block, group, or their interaction were found. Moreover, robust positive correlations of the physiological measures between the baselines levels of the first and the second block (r= 0.95 for HR, r= 0.85 for RMSSD and r= 0.85 for SI) pointed to an individual stability of cardiac activity over the two blocks of the experiment (see Figure S1). Thus our results confirmed that participants did not show differences in the physiological indices of interest at baseline.

|  | **Group** | **Block 1**  Mean (SD) | **Block 2**  Mean (SD) | **ANOVA**  Block X Group |
| --- | --- | --- | --- | --- |
| **HR** | Affective | 83.63 (10.15) | 82.46 (8.93) | Block: F= 0.26, *p*= .612, η²< 0.01  Group: F= 2.60, *p*= .109, η²= 0.01  Interaction: F=0.05, *p*= .830, η²<0.01 |
|  | Non-affective | 80.74 (11.63) | 80.25 (11.61) |  |
| **RMSSD** | Affective | 40.47 (15.45) | 38.26 (14.78) | Block: F= 0.09, *p*= .768, η²< 0.01  Group: F= 1.87, *p*= .173, η²= 0.01  Interaction: F=0.37, *p*= .544, η²< 0.01 |
|  | Non-affective | 35.84 (16.29) | 36.48 (16.68) |  |
| **Stress index** | Affective | 13.29 (5.28) | 13.18 (4.00) | Block: F= 0.03, *p*= .854, η²< 0.01  Group: F= 0.790, *p*= .375, η²< 0.01  Interaction: F<0.01, *p*= .979, η²< 0.01 |
|  | Non-affective | 13.96 (5.35) | 13.80 (4.86) |  |

*Table S1.* Descriptive statistics and analysis of baseline physiological measures (HR, RMSSD and SI).


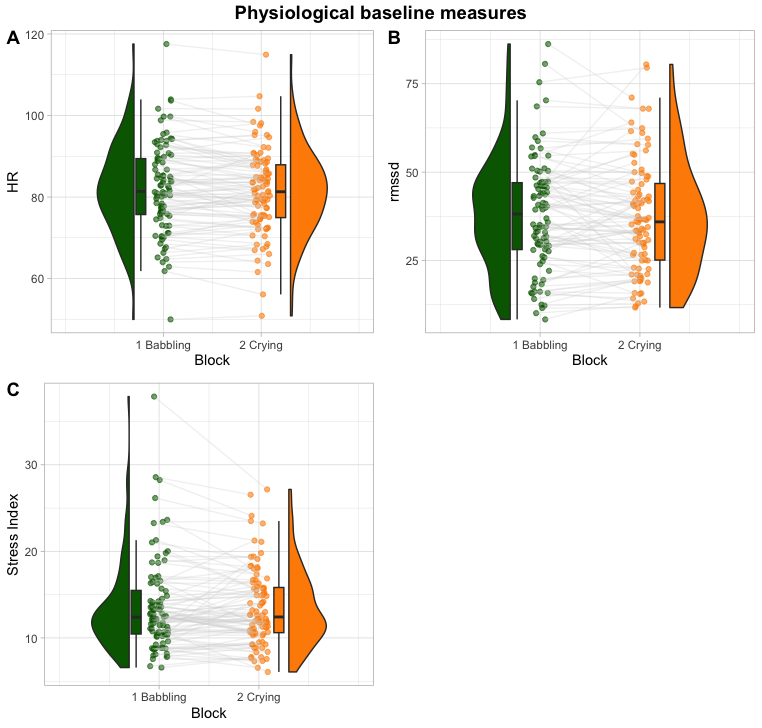


*Figure S1.* Plot of individual baseline levels and distributions of HR (A), RMSSD (B) and SI (C) in the first block (green) including the babbling video and in the second block (orange) including the crying video. Dots represent single observations; bars indicate the distribution (nparticipants = 92; nobservations = 184 for each variable).

1. **Model comparison analyses on Valence**

| Tested  models | Variables | AIC | ΔAIC | wAIC | cond R^2^ | marg R^2^ |
| --- | --- | --- | --- | --- | --- | --- |
| Model 0 | Random effect of Participants | 1932.6 |  | 0.000 | 0.024 | 0.000 |
| Model 1a | + Phase | 1911.6 | 16.49 | 0.000 | 0.075 | 0.043 |
| Model 1b | + Block | 1842.6 | 70.79 | 0.000 | 0.201 | 0.147 |
| Model 1c | + Group | 1923.6 | -80.90 | 0.000 | 0.026 | 0.021 |
| Model 2a | + Phase + Block | 1815.9 | 102.62 | 0.000 | 0.251 | 0.190 |
| Model 2b | + Phase + Group | 1902.6 | -86.13 | 0.000 | 0.077 | 0.063 |
| Model 2c | + Block + Group | 1833.7 | 70.79 | 0.000 | 0.202 | 0.168 |
| Model 3a | + Phase × Block | 1672.5 | 154.99 | 0.000 | 0.455 | 0.360 |
| Model 3b | + Phase × Group | 1903.8 | -228.69 | 0.009 | 0.081 | 0.068 |
| Model 3c | + Block × Group | 1835.2 | 70.96 | 0.000 | 0.202 | 0.168 |
| Model 4a | + Phase × Block + Group | 1663.6 | 164.12 | 0.790 | 0.456 | 0.380 |
| Model 4b | + Phase × Group + Block | 1807.5 | -142.31 | 0.000 | 0.257 | 0.214 |
| Model 4c | + Block × Group + Phase | 1808.4 | -0.11 | 0.000 | 0.253 | 0.211 |
| Model 5 | + Phase × Block × Group | 1666.3 | 135.15 | 0.201 | 0.461 | 0.385 |

*Table S2* Comparison between models predicting valence modulation. Note that smaller values of AIC indicate better fitting models.

**ANOVA on model 4a**

Chisq Df Pr(>Chisq)

(Intercept) 51.87 1 < 0.001 ***

Phase 39.40 2 < 0.001 ***

Block 303.87 1 < 0.001 ***

Group 11.35 1 < 0.001 ***

Phase × Block 171.86 2 < 0.001 ***

**Contrasts:**

Phase = Video:

contrast estimate SE df t.ratio p.value

(Block 1 - Babbling) - (Block 2 - Crying) 2.6413 0.152 455 17.432 <.0001

Phase = Touch:

contrast estimate SE df t.ratio p.value

(Block 1 - Babbling) - (Block 2 - Crying) 0.6087 0.152 455 4.017 0.0001

Phase = Recovery:

contrast estimate SE df t.ratio p.value

(Block 1 - Babbling) - (Block 2 - Crying) -0.0543 0.152 455 -0.359 0.7200

*Table S3* ANOVA and post-hoc test on model 4a. Please note that p-value adjusted for multiple comparisons using Bonferroni correction, p < 0.0167.


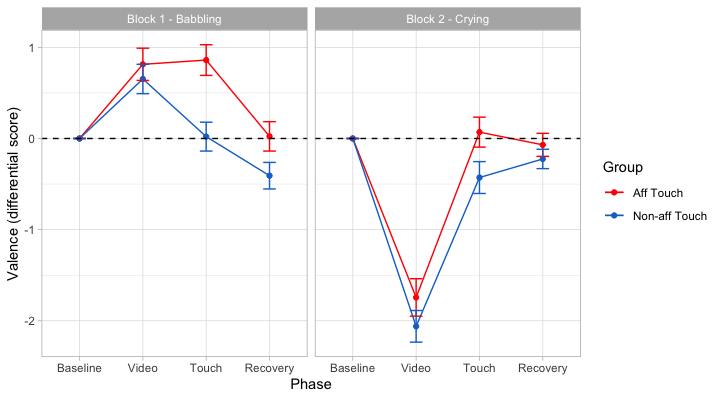


*Figure S2* Plots showing the modulation of valence across the phases of the two experimental blocks and between the two groups.

1. **Model comparison analyses on Arousal**

| Tested  models | Variables | AIC | ΔAIC | wAIC | cond R^2^ | marg R^2^ |
| --- | --- | --- | --- | --- | --- | --- |
| Model 0 | Random effect of Participants | 1950.5 |  | 0.000 | 0.162 | 0.000 |
| Model 1a | + Phase | 1831.9 | 113.52 | 0.000 | 0.357 | 0.163 |
| Model 1b | + Block | 1887.4 | -53.10 | 0.000 | 0.272 | 0.092 |
| Model 1c | + Group | 1852.4 | -64.14 | 0.000 | 0.164 | 0.000 |
| Model 2a | + Phase + Block | 1746.9 | 198.84 | 0.000 | 0.467 | 0.255 |
| Model 2b | + Phase + Group | 1833.8 | -85.32 | 0.000 | 0.359 | 0.163 |
| Model 2c | + Block + Group | 1889.3 | -53.10 | 0.000 | 0.274 | 0.092 |
| Model 3a | + Phase × Block | 1707.1 | 175.09 | 0.722 | 0.514 | 0.295 |
| Model 3b | + Phase × Group | 1835.9 | -126.32 | 0.009 | 0.360 | 0.165 |
| Model 3c | + Block × Group | 1891.3 | -52.03 | 0.000 | 0.274 | 0.092 |
| Model 4a | + Phase × Block + Group | 1709.1 | 174.63 | 0.271 | 0.515 | 0.294 |
| Model 4b | + Phase × Group + Block | 1750.5 | -40.97 | 0.000 | 0.470 | 0.256 |
| Model 4c | + Block × Group + Phase | 1750.8 | 0.74 | 0.000 | 0.468 | 0.254 |
| Model 5 | + Phase × Block × Group | 1716.3 | 28.36 | 0.007 | 0.515 | 0.295 |

*Table S4* Comparison between models predicting arousal modulation. Note that smaller values of AIC indicate better fitting models.

**ANOVA on model 3a**

Chisq Df Pr(>Chisq)

(Intercept) 8.012 1 < 0.001 **

Phase 25.544 2 < 0.001 ***

Block 129.833 1 < 0.001 ***

Phase × Block 45.458 2 < 0.001 ***

**Contrasts:**

Phase = Video:

contrast estimate SE df t.ratio p.value

(Block 1 - Babbling) - (Block 2 - Crying) -1.696 0.149 455 -11.394 <.0001

Phase = Touch:

contrast estimate SE df t.ratio p.value

(Block 1 - Babbling) - (Block 2 - Crying) -0.489 0.149 455 -3.287 0.0011

Phase = Recovery:

contrast estimate SE df t.ratio p.value

(Block 1 - Babbling) - (Block 2 - Crying) -0.446 0.149 455 -2.995 0.0029

*Table S5* ANOVA and post-hoc test on model 3a. Please note that p-value are adjusted for multiple comparisons using Bonferroni correction, p < 0.0167.


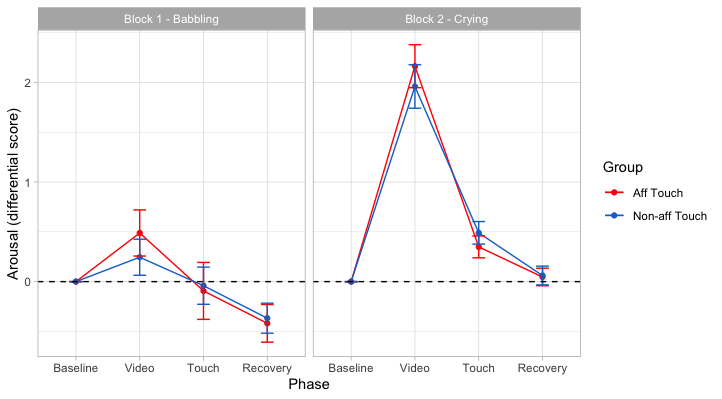


*Figure S3* Plots showing the modulation of arousal across the phases of the two  experimental blocks and between the two groups.

1. **Model comparison analyses on heart rate (HR)**

| Tested  models | Variables | AIC | ΔAIC | wAIC | cond R^2^ | marg R^2^ |
| --- | --- | --- | --- | --- | --- | --- |
| Model 0 | Random effect of Participants | 3004.1 |  | 0.000 | 0.143 | 0.000 |
| Model 1a | + Phase | 2845.8 | 156.93 | 0.043 | 0.397 | 0.212 |
| Model 1b | + Block | 3004.7 | -158.10 | 0.000 | 0.145 | 0.002 |
| Model 1c | + Group | 3001.9 | 3.34 | 0.000 | 0.145 | 0.013 |
| Model 2a | + Phase + Block | 2845.7 | 153.85 | 0.044 | 0.399 | 0.214 |
| Model 2b | + Phase + Group | 2843.6 | 3.09 | 0.127 | 0.398 | 0.224 |
| Model 2c | + Block + Group | 3002.5 | -158.10 | 0.000 | 0.148 | 0.015 |
| Model 3a | + Phase × Block | 2843.8 | 158.25 | 0.115 | 0.405 | 0.220 |
| Model 3b | + Phase × Group | 2843.7 | 1.15 | 0.123 | 0.402 | 0.228 |
| Model 3c | + Block × Group | 3004.5 | -160.62 | 0.000 | 0.147 | 0.015 |
| Model 4a | + Phase × Block + Group | 2841.6 | 161.65 | 0.343 | 0.407 | 0.232 |
| Model 4b | + Phase × Group + Block | 2843.6 | -1.93 | 0.129 | 0.404 | 0.230 |
| Model 4c | + Block × Group + Phase | 2845.6 | -2.88 | 0.048 | 0.400 | 0.226 |
| Model 5 | + Phase × Block × Group | 2846.6 | 5.69 | 0.029 | 0.410 | 0.236 |

*Table S6* Comparison between models predicting heart rate modulation. Note that smaller values of AIC indicate better fitting models.

**ANOVA on model 4a**

Chisq Df Pr(>Chisq)

(Intercept) 4.415 1 0.036 *

Phase 131.932 2 < 0.001 ***

Block 7.187 1 0.007 **

Group 4.181 1 0.041 *

Phase × Block 5.898 2 0.052 .

**Contrasts:**

Phase = Video:

contrast estimate SE df t.ratio p.value

(Block 1 - Babbling) - (Block 2 - Crying) 1.141 0.426 455 2.681 0.0076

Phase = Touch:

contrast estimate SE df t.ratio p.value

(Block 1 - Babbling) - (Block 2 - Crying) -0.303 0.426 455 -0.713 0.4763

Phase = Recovery:

contrast estimate SE df t.ratio p.value

(Block 1 - Babbling) - (Block 2 - Crying) 0.224 0.426 455 0.526 0.5992

*Table S7* ANOVA and post-hoc test on model 4a. Please note that p-value are adjusted for multiple comparisons using Bonferroni correction, p < 0.0167.


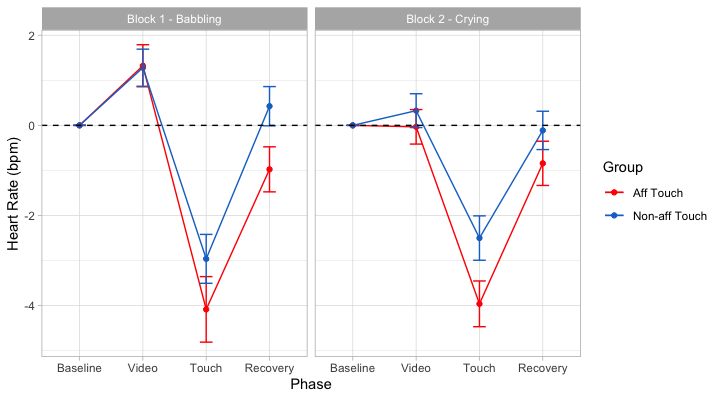


*Figure S4* Plots showing the modulation of heart rate (HR) across the phases of the two  experimental blocks and between the two groups.

1. **Model comparison analyses on heart rate variability (HRV - RMSSD index)**

| Tested  models | Variables | AIC | ΔAIC | wAIC | cond R^2^ | marg R^2^ |
| --- | --- | --- | --- | --- | --- | --- |
| Model 0 | Random effect of Participants | 3948.1 |  | 0.000 | 0.172 | 0.000 |
| Model 1a | + Phase | 3925.1 | 25.63 | 0.028 | 0.218 | 0.039 |
| Model 1b | + Block | 3943.8 | -20.20 | 0.000 | 0.183 | 0.009 |
| Model 1c | + Group | 3949.5 | -4.86 | 0.000 | 0.175 | 0.002 |
| Model 2a | + Phase + Block | 3920.3 | 30.79 | 0.298 | 0.229 | 0.049 |
| Model 2b | + Phase + Group | 3926.4 | -5.16 | 0.014 | 0.221 | 0.041 |
| Model 2c | + Block + Group | 3945.1 | -20.20 | 0.000 | 0.185 | 0.012 |
| Model 3a | + Phase × Block | 3921.3 | 29.67 | 0.181 | 0.233 | 0.053 |
| Model 3b | + Phase × Group | 3927.8 | -5.56 | 0.007 | 0.224 | 0.045 |
| Model 3c | + Block × Group | 3944.1 | -20.60 | 0.000 | 0.190 | 0.016 |
| Model 4a | + Phase × Block + Group | 3922.7 | 26.72 | 0.094 | 0.235 | 0.055 |
| Model 4b | + Phase × Group + Block | 3923.1 | -0.39 | 0.077 | 0.234 | 0.054 |
| Model 4c | + Block × Group + Phase | 3920.4 | -0.29 | 0.287 | 0.236 | 0.055 |
| Model 5 | + Phase × Block × Group | 3926.5 | 12.60 | 0.014 | 0.242 | 0.063 |

*Table S8* Comparison between models predicting heart rate modulation. Note that smaller values of AIC indicate better fitting models.

**ANOVA on model 4a**

Chisq Df Pr(>Chisq)

(Intercept) 28.685 1 < 0.001 ***

Phase 28.092 2 < 0.001 ***

Block 6.750 1 0.009 **

**Contrasts:**

contrast estimate SE df t.ratio p.value

Video - Touch -4.15 0.811 457 -5.116 <.0001

Video - Recovery -3.05 0.811 457 -3.758 0.0006

Touch - Recovery 1.10 0.811 457 1.358 0.3641

*Table S9* ANOVA and post-hoc test on model 4a. Please note that p-value are adjusted for multiple comparisons using Bonferroni correction, p < 0.0167.


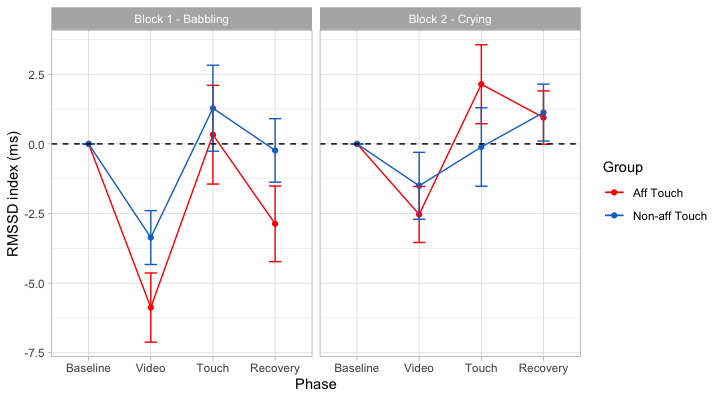


*Figure S5* Plots showing the modulation of heart rate variability (RMSSD index) across the phases of the two  experimental blocks and between the two groups.

1. **Model comparison analyses on stress index (SI)**

| Tested  models | Variables | AIC | ΔAIC | wAIC | cond R^2^ | marg R^2^ |
| --- | --- | --- | --- | --- | --- | --- |
| Model 0 | Random effect of Participants | 2710.8 |  | 0.000 | 0.198 | 0.000 |
| Model 1a | + Phase | 2684.0 | 24.86 | 0.025 | 0.248 | 0.043 |
| Model 1b | + Block | 2706.8 | -22.12 | 0.000 | 0.207 | 0.009 |
| Model 1c | + Group | 2711.7 | -3.98 | 0.000 | 0.200 | 0.004 |
| Model 2a | + Phase + Block | 2679.6 | 29.17 | 0.216 | 0.258 | 0.052 |
| Model 2b | + Phase + Group | 2684.9 | -4.31 | 0.015 | 0.251 | 0.047 |
| Model 2c | + Block + Group | 2707.8 | -22.12 | 0.000 | 0.210 | 0.012 |
| Model 3a | + Phase × Block | 2683.2 | 23.69 | 0.037 | 0.258 | 0.052 |
| Model 3b | + Phase × Group | 2682.2 | 1.91 | 0.059 | 0.261 | 0.056 |
| Model 3c | + Block × Group | 2708.9 | -26.52 | 0.000 | 0.211 | 0.013 |
| Model 4a | + Phase × Block + Group | 2684.1 | 23.36 | 0.023 | 0.260 | 0.056 |
| Model 4b | + Phase × Group + Block | 2677.8 | 6.28 | 0.539 | 0.270 | 0.064 |
| Model 4c | + Block × Group + Phase | 2681.6 | -4.46 | 0.074 | 0.226 | 0.056 |
| Model 5 | + Phase × Block × Group | 2686.1 | 0.63 | 0.009 | 0.270 | 0.066 |

*Table S10* Comparison between models predicting stress index modulation. Note that smaller values of AIC indicate better fitting models.

**ANOVA on model 4b**

Chisq Df Pr(>Chisq)

(Intercept) 12.110 1 < 0.001 ***

Phase 25.214 2 < 0.001 ***

Group 0.005 1 0.943

Block 6.407 1 0.011 *

Phase × Group 6.775 2 0.034 *

**Contrasts:**

Phase = Video:

contrast estimate SE df t.ratio p.value

(Aff Touch) - (Non-aff Touch) 0.0328 0.461 246 0.071 0.943

Phase = 2_Touch:

contrast estimate SE df t.ratio p.value

(Aff Touch) - (Non-aff Touch) -1.1358 0.461 246 -2.463 0.015

Phase = 3_Recovery:

contrast estimate SE df t.ratio p.value

(Aff Touch) - (Non-aff Touch) 0.0453 0.461 246 0.098 0.921

*Table S11* ANOVA and post-hoc test on model 4b. Please note that p-value are adjusted for multiple comparisons using Bonferroni correction, p < 0.0167.


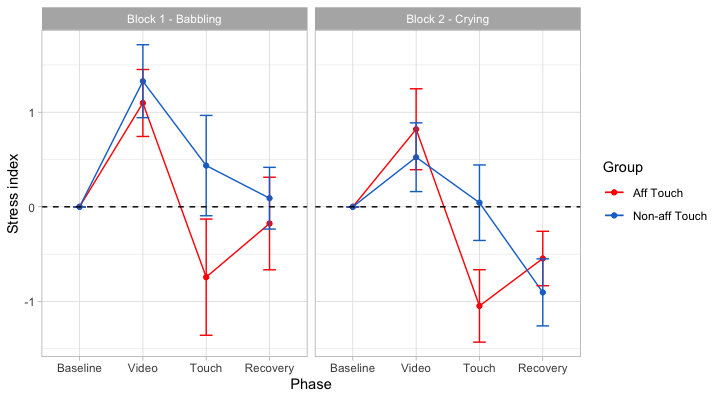


*Figure S6* Plots showing the modulation of stress index (SI) across the phases of the two  experimental blocks and between the two groups.
